# Single-stage revision in the management of prosthetic joint infections after total knee arthroplasty – A review of current concepts

**DOI:** 10.1016/j.jcot.2024.102431

**Published:** 2024-05-24

**Authors:** Tej Nikhil Pradhan, Vibhu Krishnan Viswanathan, Ravi Badge, Nikhil Pradhan

**Affiliations:** a University College London, London, UK; b Imperial College London, London, UK; c Institute of Orthopedic and Accident Surgery, Madurai, Tamil Nadu, India; d Warrington and Halton Hospitals NHS Foundation Trust, Warrington, UK; e Edge Hill University, Ormskirk, UK; f Liverpool University, Liverpool, UK; g Diploma in Sports Medicine (International Olympic Committee), UK; h University of Chester, Chester, UK

**Keywords:** (MeSH), Knee arthroplasty, Total, Prosthesis-related infection, Revision, Joint, Review literature, Treatment outcome

## Abstract

**Introduction:**

Prosthetic joint infection (PJI) is a devastating complication following total knee arthroplasty (TKA); and the gold standard surgical approach involves a two-staged, revision TKA (TSR). Owing to the newer, emerging evidence on this subject, there has been gradual shift towards a single-stage revision approach (SSR), with the purported benefits of mitigated patient morbidity, decreased complications and reduced costs. However, there is still substantial lacuna in the evidence regarding the safety and outcome of the two approaches in chronic PJI. This study aimed to comprehensively review of the literature on SSR; and evaluate its role within Revision TKA post PJI.

**Methods:**

The narrative review involved a comprehensive search of the databases (Embase, Medline and Pubmed), conducted on 20th of January 2024 using specific key words. All the manuscripts discussing the use of SSR for the management of PJI after TKA were considered for the review. Among the screened manuscripts, opinion articles, letters to the editor and non-English manuscripts were excluded.

**Results:**

The literature search yielded a total 232 studies. Following a detailed scrutiny of these manuscripts, 26 articles were finally selected. The overall success rate following SSR is reported to range from 73 % to 100 % (and is comparable to TSR). SSR is performed in PJI patients with bacteriologically-proven infection, adequate soft tissue cover, immuno-competent host and excellent tolerance to antibiotics. The main difference between SSR and TSR is that the interval between the 2 stages is only a few minutes instead of 6 weeks. Appropriate topical, intraoperative antibiotic therapy, followed by adequate postoperative systemic antibiotic cover are necessary to ascertain good outcome. Some of the major benefits of SSR over TSR include reduced morbidity, decreased complications (such as arthrofibrosis or anesthesia-associated adverse events), meliorated extremity function, earlier return to activities, mitigated mechanical (prosthesis-associated) complications and enhanced patient satisfaction.

**Conclusion:**

SSR is a reliable approach for the management of chronic PJI. Based on our comprehensive review of the literature, it may be concluded that the right selection of patients, extensive debridement, sophisticated reconstruction strategy, identification of the pathogenic organism, initiation of appropriate antibiotic therapy and ensuring adequate follow-up are the key determinants of successful outcome. To achieve this will undoubtedly require an MDT approach to be taken on a case-by-case basis.

## 1 Introduction

With the continuous growth in the volume of elderly population globally, the demand for total joint arthroplasty (TJA) has tremendously increased in the past years.^1^ Based on recent reports, the incidence of total knee arthroplasty (TKA) is anticipated to increase by an astounding rate of 276 % by the year 2030.^2^ With such a staggering rise in the number of TKAs, the rates of complications and revisions TKAs also inevitably undergo a rapid increase.^3^

Prosthetic Joint Infection (PJI) has been considered as one of the most catastrophic complications following TKA.^4^ Currently, the incidence of developing PJI after primary TKA is estimated to be around 1–2%.^5^ With a current annual rate of over 100,000 primary TKAs, it is estimated that at least 2000 patients every year may suffer from this complication within the United Kingdom (UK) alone!^6^ Given the understanding that PJIs hold an estimated two-year mortality of 7.3 %, it is crucial that effective and evidence-based management strategies are devised by clinicians to ascertain excellent healthcare delivery in these challenging situations.^7^

In a majority of situations, excepting rare scenarios of frail patients with high surgical risks, PJI essentially requires surgical intervention.^8^ The current surgical management options for PJI include debridement, antibiotics and implant retention (DAIR), single-staged (SSR) or two-staged revision arthroplasties (TSR)^8^, ^9^, ^10^, ^11^; and less commonly, salvage procedures such as arthrodesis or amputation.^12^, ^13^, ^14^ Whilst DAIR has some utility in the acute setting with stable implants; once the joint has progressed past 4 weeks of infection or there is evidence of implant loosening, its utility diminishes substantially.^8^^,^^9^^,^^15^ Although TSR has traditionally been considered the gold standard in PJI management; there has recently been a steady, global shift towards SSR, especially in the Europe, owing to the emergence of newer evidence on this subject.^16^, ^17^, ^18^, ^19^

With this background, the current study was planned to comprehensively assess the existing literature; and compare the success rates of the two interventions (SSR versus TSR), evaluate their relative indications, analyse the re-infection rates, and explore the financial burdens on healthcare systems associated with the two surgical approaches. This can potentially serve as a guidance, both to the individual clinicians considering SSR for management of their patients, as well as healthcare systems devising protocols for their concerned patient populations.

## 2 Methods

### 2.1 Literature search strategy

**Fig. 1:**
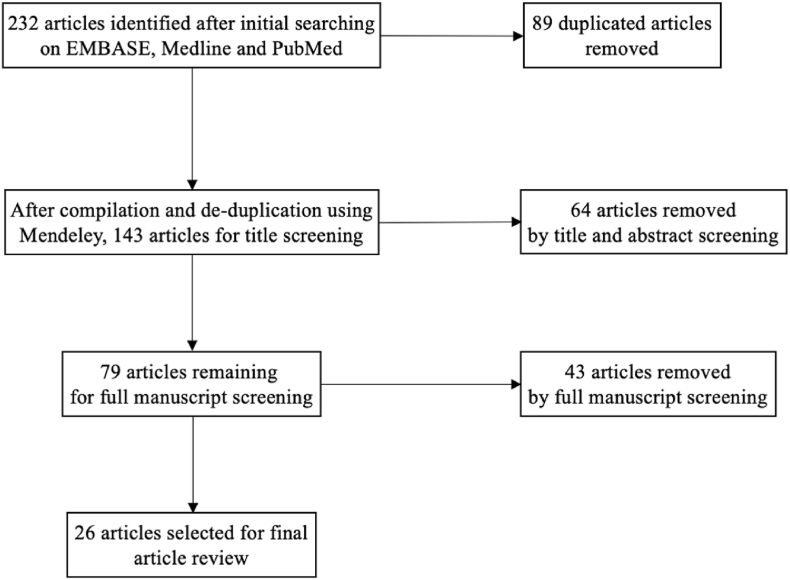
Flowchart of review process.

A rigorous literature search was conducted on January 20th, 2024 across 3 databases (Embase, Medline, PubMed) to identify articles published between 2010 and 2024. The search was conducted using a combination of Boolean Operators, namely: “periprosthetic joint infection” AND (treatment OR management) AND knee AND (“one stage” OR “single stage”) NOT fungal. The entire strategy and screening process has been presented in Fig. 1.

### 2.2 Eligibility criteria

All articles related to the implementation of SSR procedures after a primary TKA were considered within the scope of the search. Among these initially screened manuscripts; letters to the editor, opinion articles, and non-English articles were excluded.

### 2.3 Article selection and data extraction

Following the initial searches, manuscript titles and abstracts were exported to Mendeley and duplicates were removed. Subsequently, the titles and abstracts of the identified studies were screened. Following this, a second screening was conducted by reviewing the full manuscripts; and the articles were finally selected.

### 2.4 Research objectives

The main research objectives of the study included:

RO1: Defining the indications for SSR in knee PJI
RO2: Comparing the relative efficacies of SSR and TSR in the management of knee PJI
RO3: Evaluating the complication rates of SSR in knee PJI
RO4: Assessing the cost-effectiveness of SSR in knee PJI management

## 3 Results

The literature search yielded a total 232 studies. Following manual deduplication and compilation, 143 manuscripts were identified. After the screening of titles and abstract, 79 manuscripts were further considered for additional screening. Following a detailed scrutiny based on the aforementioned strategy, 26 articles were finally selected for the review.

## 4 Discussion

Deep infection is a catastrophic aftermath of TKA, and is acknowledged to impose a heavy burden on the patient, treating surgeon and entire healthcare system.^20^ It has been estimated that the management of an infected TKA can result in an expenditure, which is at least 3 to 4 times greater than that incurred following a primary TKA.^21^ The gold standard approach in PJI has traditionally remained the TSR (involving the placement of an interim antibiotic-loaded spacer), with success rates ranging between 72 % and 100 %.^22^, ^23^, ^24^ It was initially described by Insall et al.^25^ in the year 1983 in 11 patients; and all patients were eradicated of their infection at the short-term follow-up. At a mean follow-up time point of 7.5 years, the study by Goldman, Scuderi and Insall^26^ involving 64 PJI patients after TKA reported 91 % success rate. On the other hand, a recent study by Mortazavi et al.^23^ reported a failure rate of approximately 28 % following TSR at a mean follow-up of 3.8 years. There is much less data in the existing literature regarding the outcome following SSR in PJI; although a majority of available reports indicate success rates comparable to TSR. Overall, based on the published data, the success rate following SSR has been reported to range from 73 % to 100 %.^27^, ^28^, ^29^, ^30^, ^31^, ^32^, ^33^, ^34^, ^35^, ^36^, ^37^, ^38^, ^39^, ^40^, ^41^, ^42^, ^43^ It has been purported that certain factors such as duration or type of infection and type of prosthesis may influence the success rates of SSR; however, the final recommendations are still unclear.^30^ With this background, the current review was planned to comprehensively analyze status and role of SSR for the management of PJI after TKA from the data hitherto published in the existing literature; and compare the overall outcome and complication rates of SSR with the other revision and salvage surgical strategies.

### 4.1 TSR versus SSR

Although an uncommon complication, PJI is described as the most catastrophic event following a primary TKA.^44^, ^45^, ^46^, ^47^, ^48^, ^49^ Some studies have cited PJI as the most frequent cause of failure in the initial 5 years post-implantation. Surgical revision of the prosthesis is always indicated in late infections after 4 weeks of implantation.

TSR of infected total knee endoprosthesis has been associated with infection control rate of 91–96 %.^22^^,^^50^ It involves the placement of temporary, antibiotic loaded cement spacer during the interim phase.^51^^,^^52^ While the use of dynamic spacers offers the advantage of maintaining good knee mobility; static spacers can enable easier prosthesis reimplantation with minimal bone losses.^22^^,^^47^^,^^50^, ^51^, ^52^, ^53^ One-stage septic prosthesis revision has been reported only from a few select centers in a reasonably defined cohort of patients.^28^^,^^29^^,^^49^^,^^54^ The first description of SSR in infected TKA was made by von Foerster et al.,^55^ where they reported a success of 73 % in a cohort of 104 patients. Buechel et al.^29^ emphasized upon the significance of thoroughness of debridement and irrigation in determining the success of SSR. The rationale behind the concept of SSR has emerged from the fact that 90 % of the cultures procured during the second stage of revision are already infection-free.

SSR has also been described for PJI after THA. In a prospective, randomized controlled trial (INFORM trial – INFection Orthopedic Management) involving 140 adults (mean age 71 ± 9 years; 36 % female) who underwent revision total hip arthroplasty (THA),^56^ it was shown that SSR had significantly better outcome at 3 months (p = 0.003), fewer intraoperative complications (8 % in SSR versus 20 % in TSR; p = 0.01), and favorable cost-effectiveness. However, at the end of 18 months, SSR did not demonstrate any superiority over TSR for PJI in terms of patient-related outcome measures or treatment failure (p = 0.62).

A comprehensive description of the studies on SSR for PJIs following TKA has been shown in Table 1.

**Table 1:** Comprehensive review of the literature on SSR.

Goksan (1992)	18 (mean age: 61.4 years; 12 women) patients undergoing SSR Mean followup of 5 years Retrospective	Mean length of hospital stay – 26 days 1 patient - recurrent infection 1 patient - new infection 2 patients – Pain after walking 2 patients – Limited knee flexion	*Crucial factors for PJI eradication:* Aggressive debridement, use of antibiotic cement, antibiotics≥3 months *Best candidates for SSR:* gram + ve organism, no signs of systemic toxicity or gross local inflammation *Benefits of SSR:* Reduced patient morbidity, shorter hospital stay
Scott (1993)	10 patients undergoing SSR	Comparative study between SSR and TSR Mean hospital stay after SSR: 16 (Range: 14 to 21) days All patients had satisfactory functional outcome No serious complications after SSR	Recurrent infection necessitating revision TKA – 3 patients ***TSR:*** Better technique than SSR for PJI after TKA
Silva (2002)	30 reports – 37 direct exchange arthroplasty Mean followup: 4 (0.02–14) years	Infection control: 33 out of 37 (89.2 %) patients Factors associated with good outcome after SSR: gram + ve organism, absent sinus formation, use of antibiotic-impregnated bone cement for prosthesis, 12 weeks of antibiotic therapy 4 out of 2 failures of SSR: Rheumatoid arthritis patients on corticosteroids	***SSR:*** Successful in healthy host with sensitive organism and long antibiotic therapy
Buechel (2004)	22 consecutive patients Mean followup of 10.2 years (ranging between 1.4 and 9.6 years) Retrospective	Organisms cultured: Mixed in origin (Staphylococcus epidermidis and Staphylococcus aureus -most common) Infection eradication rate: 90.9 % Mean knee score: 79.5 (out of 100 points), with good to excellent results in 90.9 % of patients	***SSR:*** Physiological classification of host – Class A or B (20 patients) – Successful infection eradication in all patients Class C – 2 patients – failure (1 – death secondary to multi-organ failure; 1 – Re-revised after 6.5 years
Bauer (2006)	Multicentric retrospective study	Comparative study between SSR and TSR	No difference between TSR and SSR in the rate of infection eradication ***SSR:*** Excellent outcome in 40 % of patients ***TSR:*** Excellent outcome in 33 % of patients
Parkinson (2011)	12 patients “Two-in-one” technique	Two patients with active discharging sinus at presentation – healed well Mean followup of 2 years	No cases of recurrent infection
Whiteside (2011)	18 patients with MRSA infection Mean followup: 62 (27–96) months Retrospective Intravenous antibiotics – only for first 24 h	Single stage debridement and implant revision + intraarticular infusion of 500 mg vancomycin using Hickman catheter – one or twice a day for 6 weeks Serum vancomycin level monitoring: 3–10 microg/ml	SSR + intraarticular vancomycin infusion: Excellent technique Recurrent infection necessitating re-debridement: 1 patient No complications
Singer (2012)	63 (6 UKA, 37 primary TKA, 20 hinged knee endoprosthesis) between 2004 and 2006; mean followup: 36 months) MRSA/MRSE/unknown microorganisms Retrospectively reviewed prospective data	3/20 patients with hinged knee prosthesis and chronic infections - Recurrent infection Mean Knee Society Knee Score (At 24 months): 72 (20–98) points Oxford-12 Knee Score (At 24 months): 27 (13–44) points	***SSR:*** 95 % success rate; higher knee score than TSR ***Failure risk for SSR:*** Long-term chronic infection, hinged prosthesis
Baker (2013)	Patient-related outcome measure (PROM) for 33 patients undergoing SSR	Mean OKS: 24.9 (SSR) vs 22.8 (TSR); p > 0.05 Mean EQ5D index: 0.495 (SSR) vs 0.473 (TSR); p > 0.05 Excellent patient satisfaction rate: 66 % (SSR) vs 60 % (TSR); p > 0.05	No statistically significant difference in functional outcome between SSR and TSR
Tibrewal (2014)	50 patients (mean age: 66.8 years; 33 women; SSR between 1979 and 2010) Mean followup: 10.5 (2–24) years Retrospective	Mean interval between primary TKA and revision surgery: 2.05 years (1–8 years)	***SSR:*** 98 % success rate ***Complications after SSR:*** Recurrent infection necessitating revision TKA – 1 patient Septic episodes not requiring revision TKA – 3 patients Aseptic loosening necessitating revision TKA – 9 patients
Klatte (2014)	4 patients with fungal PJI (operated between 2001 and 2011) Mean followup: 7 (3–11) years	Mean Hospital for Special Surgery knee score increase: 75 points (70–80: p < 0.01)	***Recurrent infection necessitating revision TKA:*** 1 patient (Revision at 29 months' postoperative time point) ***SSR:*** Feasible option in fungal PJI after TKA with successful outcome
Haddad (2015)	28 (Mean age: 65 years) patients undergoing SSR (2004–2009) Mean followup: 6.5 (3–9) years	Indications for SSR: Minimal bone loss No immunocompromise Healthy soft tissue Known organism with known antibiotic sensitivity No recurrent infection in SSR Higher KSS in SSR (88 vs 76; p < 0.001)	Supported the use of SSR in selected cohort of patients Need for large, multicenter prospective study
Labruyere (2015)	9 (6 medial, 2 lateral, 1 patello-femoral) patients with infected UKA (operated between 2003 and 2010) Mean age: 67 (36–83) years Mean followup: 60 (36–96) months Retrospective single-center study	Dual intravenous antibiotic therapy for 6 weeks, followed by oral antibiotics for 6 weeks Single stage UKA to TKA revision Mean duration of infection: 9 months Oxacillin-sensitive Staphylococcus: 6 patients, Streptococcus: 1 patient, Enterococcus: 1 patient, Escherichia coli: 1 patient	No recurrent infection or revision surgery for infection No medical or antibiotic-related complications Significant improvement in International Knee Score (IKS): Knee (60–75 points) and function (50–65 points) score ***SSR of UKA to TKA:*** Identification of causative organism, synovectomy, joint excision, same-stage TKA, followed by antibiotic therapy * 3 months
Cochran (2016)	1,493,924 patients from Medicare data (2005–2011) 16,622 patients with PJI; 3069 (22.7 %) treated with SSR	SSR: 33.9 % greater adjusted risk of reinfection than TSR (p < 0.001)	TSR: Significantly higher success rate than SSR
Massin (2016)	108 patients undergoing SSR Retrospective, multicenter study comparing SSR and TSR (2005–2010) Followup >2 years	Risk factors for infection recurrence: Presence of fistula (p = 0.03) Gram -ve bacteria (p = 0.05) SSR – Better outcome (similar infection risk with greater comfort) in female patients	Routine SSR can be performed
Kunutsor (2016)	Systematic review and metaanalysis	Reinfection rate from 10 (423 patients) studies on SSR: 7.6 % (vs 8.8 % in TSR) Functional outcome – similar between SSR and TSR	SSR: As effective as TSR
Castellani (2017)	35 patients undergoing SSR Retrospective (2000–2013)	No statistically significant difference in clinical outcome between SSR (success rate: 94.2 %) and TSR (success rate: 84 %); p > 0.05 Enterococcus and peptostreptococcus – high treatment failure	Large, prospective trials necessary to recommend the best practice guidelines
Holland (2019)	Prospective study (2009–2017) 26 patients (mean age: 72.5 years, mean BMI: 33.4, median ASA physical status classification: 2) with significant bone loss and PJI Among them, 2 patients – failed TSR; 1 patient – failed DAIR	“2-in-1” SSR in associated bone loss Functional assessments: Knee range of motion (ROM), Oxford Knee Score (OKS), American Knee Society Score (AKSS), Short Form-12 (SF-12) Mean time to revision: 3.5 (3 months–12) years 6 patients: active discharging sinus (preoperatively); 4 patients: no positive microbiological culture (preoperatively)	Recurrent infection (requiring long-term antibiotic suppression): 1 patient ***Significant improvements in*** Pain component of AKSS score: 4.3 (preoperative) to 32.4 (postoperative) Functional component of AKSS score: 10.7 (preoperative) to 15.7 (postoperative) Mean knee extension: 18.5 (preoperative) to 6.9 (postoperative) Mean total ROM: 69.2 (preoperative) to 90.3 (postoperative)
Yaghmour (2019)	PRISMA systematic review (Risk bias: ROBINS-I tool; Quality assessment: GRADE criteria) 16 studies (3645 TKA)	SSR: Satisfactory outcome, low re-infection rate, good functional outcome	Large, randomised trials are needed to ascertain if strict patient selection criteria are needed for choosing patients for SSR
Kildow (2020)	Review	SSR: Comparable success rate between SSR and TSR Shared decision with patient – crucial	Benefits of SSR: Better functional ability of patient; decrease burden to health care system
Lum (2020)	Review	Success of SSR: Careful patient selection, identification of organism and precision surgical technique 3 basic principles: Bacterial sensitivity, radical debridement, local and systemic antibiotic delivery	Future randomised studies to assess the role in culture-ve organisms, and use of cementless prosthesis
Palmer (2020)	Review and expert opinion		***SSR:*** Well-suited for susceptible organisms and in patients without comorbidities or unable to undergo 2 surgeries
Brunt (2021)	24 patients (mean age: 72.7 years, mean BMI: 33.3, median ASA physical status classification: 2) with significant bone loss and PJI Prospective Minimum followup: 5 years	Mean time to revision: 3 (10 months–8.3) years Functional assessments: Oxford Knee Score (OKS), American Knee Society Score (AKSS) 6 patients: active discharging sinus (preoperatively); 2 patients: no positive microbiological culture (preoperatively)	Recurrent infection: 2 patients (1 patient - long-term antibiotic suppression; 1 patient - DAIR) Mean AKSS score: 27.1 (preoperative) to 80.3 (2 years; p < 0.001) to 74.1 (5 years; p = 0.081) Mean 2-year vs 5-year OKS score: 33 vs 36.17 (p = 0.081) 2-in-1 revision in PJI with bone loss: Sustained functional improvement and infection clearance until 5 years
Mu (2023)	Review	SSR from chronic PJIs after knee and hip arthroplasties	***SSR:*** Reliable technique for chronic PJI Determinants of success: Careful patient selection, radical debridement, excellent reconstruction and appropriate antibiotic therapy
Peddada (2023)	Review	24 studies – 147 SSR after TKA (1984–2019) Revision surgery rate: 11.8 % Revision due to infection: 3.3 % Revision due to aseptic loosening: 8.8 % Revision due to instability and fracture (combined) < 3 % Mortality rate <3 %	***SSR following TKA:*** High survivorship and low mortality
Goud (2023)	Systematic review and metaanalysis (2015–2020)	Reinfection rate: 12.7 % (SSR) vs 16.2 % (TSR)	Similar reinfection rates between SSR and TSR

Abbreviations: SSR: Single stage revision,TSR: Two-staged revision, PJI: Prosthetic joint infection, TKA: Total knee arthroplasty, DAIR: Debridement, antibiotics and Implant retaining, UKA: Unicompartmental knee arthroplasty, ROM: Range of motion, MRSA: Methicillin resistant staphyloscoccus aureus; MRSE: Methicillin-resistant staphylococcus epidermidis.

### 4.2 Indications for SSR

Positive cultures for fastidious organisms such as MRSA and MRSE have been considered as contraindications for planning SSR across a majority of published studies, since the infection control of PJIs is generally much lower for these pathogens as compared to other bacteria.^30^ In a retrospective by Tibrewal et al.,^57^ SSR was performed only in PJI patients where the culture/sensitivities of the tissues were available, organisms were identified (bacteriologically-proven infection); and soft tissue cover over the knee was intact. The study by Buechel et al.^29^ showed that the physiological status of the individual is a crucial parameter in determining the success of reimplantation. Overall, immuno-competent patients with susceptible organisms and excellent tolerance to antibiotics can be good candidates for successful outcome.^29^ In this study, the patients were stratified into 3 groups on the basis of their physiological status^58^: “A” host – good immune system and delivery, “B” host – compromised locally or systemically; and “C” host – poor health status and not a surgical candidate. The outcome following SSR was substantially poorer in host type C, as compared to A and B. The overall indications and contraindications of SSR have been presented in Table 2.

**Table 2:** Indications and contraindications of SSR.

Indications	Contraindications
**Systemic health of patients**	**Systemic health of patients**
No active systemic sepsis	Active systemic infection or concurrent sepsis
Non-severely immunocompromised host	Immunocompromised host
	Major systemic illness
**Local skin and soft tissue**	**Local skin and soft tissue**
Good soft tissue	Infection involving neurovascular bundles
No substantial bone defects or losses	Peripheral vascular disease
Previous revision surgeries ≤2 times	Previous failed revision surgeries on >2 occasions
	Active sinus tract
	Previous TSR
	Significant bone defects
**Pathogen**	**Pathogen**
Known pathogens	Fastidious or difficult-to-treat pathogens
Good susceptibility to antibiotics	Unidentified pathogens
	Polymicrobial disease
**Antibiotic therapy**	**Treatment-related**
Antibiotic-laden cement application	Inability to provide local antibiotic therapy
No major bone graft needed for reconstruction	Inability to perform radical tissue debridement
Good oral bioavailability of antibiotics	

Abbreviations: SSR: Single stage revision, TSR: Two stage revision.

**Table 3:** Steps of “two-in-one” revision TKA.

**Step 1**	Preoperative identification of the pathogen (possibly through arthroscopic biopsy)
**Step 2**	Thorough intraoperative debridement, irrigation and implant removal
**Step 3**	Take break for a few minutes
**Step 4**	Rescrubbing, redraping and new instrumentation set to be utilised
**Step 5**	The implantation of modular revision prosthesis with additional antibiotics (tobramycin, gentamycin, vancomycin) in the bone cement
**Step 6**	Immediate starting of postoperative antibiotic therapy (based on recommendations from microbiology and infectious diseases team)
**Step 7**	Antibiotic therapy to be continued for a minimum of 6 weeks postoperatively (parenteral or oral) based on the final microbiology report
**Step 8**	Immediate weight bearing and range of motion exercises allowed

Abbreviations: TKA: Total knee arthroplasty.

### 4.3 Operative procedure for SSR (Table 3)

The operative procedure for SSR was initially described by Goksan et al.^28^^,^^59^ and is essentially similar to the delayed reimplantation technique in terms of the technique. A majority of the studies published since then have also adopted a similar approach. Prior to definitive surgery, an attempt is made to identify and culture the pathogen by needle aspiration or arthroscopic biopsy. Antibiotic therapy is withheld preoperatively. The knee is approach through the previous incision; and swabs or tissues samples are obtained from the joint. The components (including the entire cement mantle) are then removed and sent for culture. Following this, the interface membrane is removed; and sent for histological and bacteriological evaluation. The joint is then thoroughly debrided and all tissues with questionable viability are excised. The joint is then washed extensively with copious volumes of normal saline; and packed with povidone-iodine-soaked swabs. The wound edges are then approximated with a few sutures, temporary compressive dressing applied, appropriate antibiotics are administered intravenously; and the tourniquet is deflated for 30 min.

The operating team then changes the gloves and gowns; the patient is re-draped completely; and a new set of instruments is arranged for the next phase of the procedure. The tourniquet is inflated, joint washed with normal saline; and cultures are sent from the bone surfaces. The new implants are then inserted (along with antibiotic-laden PMMA cement in cemented prostheses or after being dusted with antibiotic powder in uncemented prostheses). Two suction drains are then inserted into the joint and the wound is closed in layers. The joint is initially splinted, followed by gradual range of motion (ROM) exercises.

### 4.4 Antibiotic protocol

The antibiotics are administered intravenously initially (based upon the antibiotic sensitivity). After 2 weeks, the antibiotic therapy is shifted to an oral regimen, which is then usually continued for 3 months. Buechel et al.^29^ recommended a longer antibiotic regimen involving organism-specific, intravenous antibiotic therapy for 4–6 weeks, followed by oral antibiotics for an additional period of 3–6 months.

Mu et al. discussed the 5D's in the eradication of infection: “diagnosis of pathogen, dosage, duration, duality and delivery”.^43^^,^^60^ It has been recommended that in view of the biofilm remnant in such cases, antibiotics must be administered at the dosage of minimum biofilm eradication concentrations (MBECS). Such concentrations are approximately 100 to 1000 times greater that the minimum inhibitory concentrations (MIC) necessary to eradicate planktonic organisms.^43^^,^^61^ Since it is not feasible to administer such high dosages through intravenous antibiotic regimens alone (in view of systemic toxicity); the use of topical or local antibiotic delivery strategies (such as antibiotic beads, sponges or powder) is recommended. Some recommendations regarding the antibiotic regimen, as purported by Mu et al.,^43^ have been presented in Table 4.

**Table 4:** Protocol for antibiotic therapy.

	Positive bacterium culture	Negative bacterium culture
Intravenous preoperative	Pathogen-sensitive antibiotic therapy	Vancomycin – 1 g
Topical intraoperative	Vancomycin powder (0.5 g)/Meropenem powder (0.5 g)	Vancomycin powder (0.5 g)
Intraveneous postoperative	Pathogen-sensitive antibiotics * 2 weeks	Vancomycin every 12 h for 2 weeks
Intraarticular infusion postoperative	Multidrug-resistant bacteria, fungi, polymicrobial infection: pathogen-sensitive antibiotic * 12–14 days	Vancomycin (0.5 g) – Morning Meropenem (0.5 g) – Evening * 12–14 days
Oral postoperative	Following parenteral and topical antibiotic therapy, quinolones and rifampicin may be utilised as oral switch therapy* 31 days; until erythrocyte sedimentation rate (ESR) and *C*-reactive proteins (CRP) decrease or return to normal levels	

### 4.5 Outcome and factors determining the overall results

Overall, the primary outcome measure employed in the studies for evaluating the results following SSR is the rate of recurrence of infection.^16^^,^^27^^,^^33^^,^^43^^,^^50^^,^^56^^,^^57^^,^^61^, ^62^, ^63^, ^64^, ^65^, ^66^ Studies have also evaluated the rates of surgical revision secondary to infection or non-infective causes. Peddada et al.^16^ evaluated the survivorship of the TKA prostheses. Among the parameters employed for functional evaluation of patients; range of motion (ROM), and functional knee scores [like American Knee Scociety Score (AKSS), Oxford Knee Score (OKS) or International Knee Society scores] have been assessed in many of the reviewed studies.^30^^,^^38^^,^^39^^,^^57^^,^^64^^,^^66^ In general, a majority of the studies hitherto published, have demonstrated comparable success (or infection eradication) rates following SSR, as reported with TSR.^4^^,^^27^^,^^30^^,^^31^^,^^37^^,^^38^^,^^40^^,^^42^^,^^55^^,^^56^^,^^62^^,^^64^^,^^65^^,^^67^

In a retrospective study by Tibrewal et al.^57^ involving 50 (33 women; mean age: 66.8 years) consecutive patients undergoing SSR for established deep infection after TKA, the overall success rate was reported to be 98 %. Based on their analysis, it was concluded that SSR produces comparable results to the TSR, with the additional benefits of reduced morbidity and inconvenience to patients, as well as mitigated health care expenditure.

In the retrospective series published by Buechel et al.,^29^ 22 consecutive patients undergoing SSR for PJI following TKA were assessed at the mean followup time point of 10.2 years (ranging between 1.4 and 9.6 years). The organisms cultured were mixed in origin, with Staphylococcus epidermidis and Staphylococcus aureus being the most commonly isolated. The overall infection eradication rate following SSR was 90.9 % in this series; and the mean knee score at the final followup was 79.5 (out of 100 points), with good to excellent results reported in 90.9 % of patients.

Based on the review by Kunutsor et al.^38^ comparing the reinfection rates after SSR and TSR, it was concluded that both the approaches were effective in treating infected knee prosthesis [reinfection rate of 7.6 % (SSR) versus 8.8 % (TSR)]. In the largest study on SSR based on Medicare Registry in the United States, involving 3069 patients (by Cochran et al.),^67^ the reinfection rates after SSR were 24.6 % and 38.25 % at the end of 1 and 6 postoperative years, respectively. These rates were significantly higher than the rates after TSR (19 % and 29 % at 1 and 6 years, respectively).

Elderly age has been discussed as a crucial factor determining the rate of reinfection after SSR. While Massin et al.^63^ reported higher infection rate (23 %) in the elderly cohort; such an association was not observed by Kunutsor et al.^38^ and Cochran et al.^67^ Obstructive sleep apnea (OSA) and body mass index (BMI) greater than 30 kg/m^2^ have been associated with higher reinfection rates following SSR.^30^^,^^39^^,^^43^ Local factors such as previous joint surgery, rotating hinge prosthesis, gram -ve bacterial infection, fungal pathogen and presence of sinus or fistula have been correlated with the higher occurrence of reinfections.^39^ In addition, in a review by Yaghmour et al.,^39^^,^^63^ systemic factors such as diabetes mellitus, smoking, rheumatoid arthritis, depression and steroid use have been correlated with enhanced rates of reinfection after SSR.

All the reviewed studies in our review have reported successful outcome after SSR.^16^^,^^22^^,^^34^^,^^39^, ^40^, ^41^, ^42^, ^43^^,^^57^^,^^62^^,^^66^ Significantly better postoperative improvements in KSS following SSR, as compared with TSR, have been reported by Singer et al.^30^ and Haddad et al.^64^ In the studies by Singer et al.,^30^ Tibrewal et al.,^57^ and Baker et al.,^66^ significantly better postoperative OKS scores were observed following SSR (as compared to TSR). In a recent systematic review, Yaghmour et al.^39^ observed substantial heterogeneity in the functional scores reported in the literature heretofore published; and highlighted the need for better quality, randomized, prospective trials on this subject.

### 4.6 Purported benefits over TSR

Parkinson et al.^27^ described the SSR as a “two-in-one” technique, wherein the main difference from TSR is that the interval between the 2 stages is only a few minutes instead of 6 weeks. SSR can potentially eliminate the pitfalls of a traditional TSR such as knee stiffness and arthrofibrosis. The fact that a SSR can potentially save the patient from undergoing the hassles of a second surgery, such as additional expenditure, added morbidity and substantial inconvenience, has been highlighted in this study.

In a recently-published review by Mu et al.,^43^ SSR has been described as a patient-centered solution with a wide spectrum of advantages including shorter hospitalization, mitigated surgical and anesthetic risks, better extremity function and substantially earlier return to activities. They also described a lower rate of prosthesis-related mechanical complications such as prosthetic dislocations and peri-prosthetic fractures, in comparison with TSR. They highlighted that SSR is associated with lower mortality and morbidity; as well better patient satisfaction rates. They observed that SSR is gaining popularity among the patients as well as surgeons in view of the mitigated complication risks with non-inferior infection control rates, as compared to TSR.

### 4.7 Complication rates

In a recent review by Peddada et al.,^16^ at a weighted mean followup time point of 69.8 months (mean patient age: 66.3 years; 55 % males; 24 studies; 147 patients), the overall revision surgical rate was 11.8 %. In this series, the rate of revision due to aseptic loosening following SSR (8.8 %) was substantially greater than the revision rates due to recurrent infection (3.3 %) or secondary to mechanical complications (fractures or dislocations; <3 %).

At a mean followup time point of 10.5 years, in the study by Tibrewal et al.,^57^ 9 patients underwent revision TKA following aseptic implant loosening; while 3 others developed a further septic episode (none of whom required a revision surgery). The overall mortality rate following SSR is reported to be less than 3 %.

### 4.8 Limitations

Our narrative review has diverse limitations, inherent to most of the non-systematic reviews. There was no definitive strategy employed to evaluate the methodological quality of the included studies. A majority of the studies have employed small sample sizes and are based on retrospective/non-randomized data. There is substantial heterogeneity in the treatment protocols used, antibiotic regimens followed and followup parameters evaluated. Despite these shortcomings, our review comprehensively summarizes our current understanding regarding the exact role and status of SSR in the management of PJI after TKA.

## 5 Conclusion

SSR is a reliable approach for the management of chronic PJI. Meticulous treatment protocols and experienced, dedicated multidisciplinary teams are necessary to ascertain the success of this strategy. Based on our comprehensive review of the literature, it may be concluded that the right selection of patients, extensive debridement, sophisticated reconstruction strategy, identification of the pathogenic organism, initiation of appropriate antibiotic therapy (in concordance with the recommendations of infectious disease and microbiologist teams) and ensuring adequate follow-up are the key determinants of successful outcome following SSR in PJI. To achieve this will undoubtedly require an MDT approach to be taken on a case-by-case basis.

## CRediT authorship contribution statement

**Tej Nikhil Pradhan:** Conceptualization, Data curation, Investigation, Methodology, Project administration, Validation, Visualization, Writing – original draft, Writing – review & editing. **Vibhu Krishnan Viswanathan:** Data curation, Investigation, Methodology, Project administration, Validation, Visualization, Writing – original draft, Writing – review & editing. **Ravi Badge:** Supervision, Project administration, Writing – review & editing. **Nikhil Pradhan:** Conceptualization, Supervision, Project administration, Writing – review & editing.

## Declaration of competing interest

The authors declare that they have no known competing financial interests or personal relationships that could have appeared to influence the work reported in this paper.
